# How organizational escalation prevention potential affects success of implementation of innovations: electronic medical records in hospitals

**DOI:** 10.1186/s13012-016-0435-1

**Published:** 2016-05-20

**Authors:** Mattijs S. Lambooij, Ferry Koster

**Affiliations:** 1 Department of Quality in Health Care and Health Economics, National Institute of Public Health and the Environment, A van Leeuwenhoeklaan 9, 3720 BA Bilthoven, The Netherlands; 2 Department of Sociology, Erasmus University Rotterdam, Burgemeester Oudlaan 50, 3000 DR Rotterdam, The Netherlands; 3 TIAS School for Business and Society, Warandelaan 2, Tias Building, 5037 AB Tilburg, The Netherlands

**Keywords:** Technology implementation, Escalation of commitment, Escalation prevention potential, Electronic medical record, Perceived added value

## Abstract

**Background:**

Escalation of commitment is the tendency that (innovation) projects continue, even if it is clear that they will not be successful and/or become extremely costly. Escalation prevention potential (EPP), the capability of an organization to stop or steer implementation processes that do not meet their expectations, may prevent an organization of losing time and money on unsuccessful projects. EPP consists of a set of checks and balances incorporated in managerial practices that safeguard management against irrational (but very human) decisions and may limit the escalation of implementation projects. We study whether successful implementation of electronic medical records (EMRs) relates to EPP and investigate the organizational factors accounting for this relationship.

**Methods:**

Structural equation modelling (SEM), using questionnaire data of 427 doctors and 631 nurses who had experience with implementation and use of EMRs in hospitals, was applied to study whether formal governance and organizational culture mediate the relationship between EPP and the perceived added value of EMRs.

**Results:**

Doctors and nurses in hospitals with more EPP report more successful implementation of EMR (in terms of perceived added value of the EMR). Formal governance mediates the relation between EPP and implementation success. We found no evidence that open or innovative culture explains the relationship between EPP and implementation success.

**Conclusions:**

There is a positive relationship between the level of EPP and perceived added value of EMRs. This relationship is explained by formal governance mechanisms of organizations. This means that management has a set of tangible tools to positively affect the success of innovation processes. However, it also means that management needs to be able to critically reflect on its (previous) actions and decisions and is willing to change plans if elements of EPP signal that the implementation process is hampered.

**Electronic supplementary material:**

The online version of this article (doi:10.1186/s13012-016-0435-1) contains supplementary material, which is available to authorized users.

## Background

Implementation processes are prone to a phenomenon called ‘escalation of commitment’, meaning that projects continue, even if it is clear that they will not be successful and/or that the costs will be much higher than expected [1]. Famous examples of such escalating projects are the Concorde airplane, which eventually flew between the UK and France after large amounts of money were spent on its development, and the Long Island power plant that was intended to cost 75 million dollars but that ended up costing six billion dollars. Decision-makers face a dilemma when they are confronted with a failed course of action as they can accept the loss and choose a more promising strategy or stick to a plan that already consumed considerable amounts of money and time. Escalation of commitment refers to the processes through which decision-makers choose the latter even when it is more rational to stop.

There is some evidence showing that organizations possessing escalation prevention potential (EPP) may prevent escalation of commitment [2, 3] and thus avoid costly mistakes. However, it is not known whether EPP contributes to the overall success of innovation projects. This paper investigates whether there is a relationship between EPP and implementation success and, if so, whether formal governance and organizational culture mechanisms can explain this relationship.

In this paper, the focus is on a particular kind of innovation, namely the introduction of electronic medical records (EMRs) in hospitals. This study extends prior research into EMR implementation by examining the social processes explaining successful implementation. In order to do so, we rely on insights from organizational research stating that the success of innovation projects depends on the management of an organization [1, 4, 5].

The concept of EPP is based on management research showing that the success of innovation projects depends on the ability to steer or stop unsuccessful projects [4–12]. This ability is theoretically rooted in the ‘escalation of commitment’ literature [1, 4]. Basically, this literature challenges rational models of project management emphasizing that managers do not always monitor the process and outcomes of projects to make accurate adjustments [13]. In such cases, the management of an organization lacks objective information about the project and is at higher risk for escalation of commitment, which results in pushing through unsuccessful (implementation) projects.

Escalation is caused by several determinants at different levels of analysis [5] and is explained by multiple theoretical mechanisms (e.g. subjective expected utility, self-justification, framing, goal-substitution, self-presentation, agency problems, loss aversion and confirmation bias) [1]. When a management installs and maintains EPP, it means that they are willing to critically reflect on their own performance and that they are willing to change if initial ideas did not turn out the way they were planned: they will use feedback from the organization to objectively improve their own performance during an implementation process. The use of constructive criticism from objective evaluation may result in organizational governance that fosters the implementation and subsequent quality of innovations.

### Electronic medical records in hospitals

EMRs are systems to store patient information that enable the exchange of information amongst healthcare professionals, assist professionals in decision-making and can improve patient safety. While EMRs explicitly aim at improving the work performance in healthcare organizations, this does not happen automatically. Studies on implementation of EMRs show that it is difficult to determine the objective added value of an EMR [14–19], and that resistance of users [20–25] or other barriers [26–29] can block its potential benefits [30]. This implies that purely rational models of technological innovation, according to which innovations create added value for all stakeholders in an organization by definition and that do not regard change process as a part of a larger social system that needs continuous attention to be successful [31], do not suffice to understand innovation processes completely. While the literature on escalation of commitment focuses on biases hindering rational decision-making, this does not exclude the possibility that managers may be aware that they too can be a subject of psychological biases. By installing safeguards that result in a stronger tendency to resist escalation of commitment (e.g. having more EPP), they may try to minimize the likelihood of project failure. In the case of implementation of EMRs in hospitals, this implies that hospitals that possess more EPP are expected to implement their EMRs more successfully than organizations with less EPP.

### Mechanisms of escalation prevention potential

We start with the overall expectation in organizations with more ability to safeguard innovation projects against poor decision-making (because they possess more EPP); the added value of implemented innovations for its key users will be larger than in organizations with less EPP [2, 6, 10, 12]. Organizations with more EPP have clearly defined the goals of the project, evaluate the process with measurable performance indicators that are based on those goals, divide the process in smaller parts and assign people to the project with the right qualification and that have clearly defined responsibilities.

Because projects and organizations with more EPP are assumed to perform better in terms of foreseeing and preventing problems that may occur during the implementation process, the result of that process is expected to be better. The basic expectation of our theoretical model is that ‘in organizations with more escalation prevention potential, the key users perceive more added value of using the innovation.’ (hypothesis 1).

Besides assuming that this direct relationship between the prevention of escalation and the success of the implementation process, it is possible to explain this relationship using organizational theories. Here, we propose that EPP affects the success of an implementation process through an organization’s formal governance structure and its culture.

#### Formal governance

Firstly, a positive relationship between escalation prevention and implementation success may be the result of characteristics of the formal organization that enable individuals to do their job well. Several organizational characteristics are considered. The first aspect relates to the role of supportive staff (IT, administration and HRM [29, 32–34]) and particularly how well they perform. Obviously, well-functioning support staff departments provide assistance to the key users of a new technology. In organizational contexts in which these departments do not perform well, organizational members may face difficulties in learning to apply the innovation in their daily work, which hampers the full potential of the innovation. There is also a link with the EPP of the organization. As in organizations with more EPP, unsuccessful projects are disbanded more quickly, the people from support departments are less busy solving issues of problematic projects and are able to perform the assigned tasks.

The second aspect concerns the leadership style applied within the organizations. Both the reflexive leadership of managers and the possibilities for employees voice their opinions to the management of the organization are productive in that sense and stimulate individual performance [29, 35–37]. These aspects are in turn related to the extent to which organizations are able to prevent escalation of commitment as reflexive leadership and input from organizational members go hand in hand with the ability to stop and steer innovations. Together, these aspects span the formal governance relating to escalation prevention and individual work performance. Hence, hypothesis 2 reads: ‘Formal governance mediates the association between organizational EPP of the organization and perceived added value for the key users of the innovation.’ (hypothesis 2).

#### Organizational culture

In addition to the formal governance of organizations, organization culture may affect the success of implementation projects. What is known from the literature is that organizational culture can support or hinder the performance of individuals [29, 38–41]. Here, two characteristics of organizational culture are investigated, namely whether organizations have a culture of innovativeness and how open their culture is to change [42, 43]. With regard to the use of new technologies, it can be argued that working in an environment where members of the organizations value innovation, in which they are willing to try new things, and see changes as a positive thing, it is likely that there is a more positive attitude towards new technologies. And, as a result of that, these technologies will be used much more effectively than in organizations without these cultural traits. Organizational cultures develop over time, and the experiences that organizational members have with the way in which innovation projects are organized and how successful these projects are may be an important driver of cultural values concerning innovation and organizational change. Hence, in organizations with higher levels of EPP, organizational members may have learned that technological innovations can have a positive outcome. This in turn creates an atmosphere in which innovations and change are valued. The third hypothesis is therefore that ‘Organisational culture, (i.e. innovativeness of the culture (3a) and openness to change (3b)) mediates the association between organizational EPP and perceived added value for the key users of the innovation.’ (hypothesis 3).

## Methods

### Models

To test whether formal governance and organizational culture explain the association of EPP and successful implementation, we ran two structural equation (structural equation modelling (SEM)) models. In model 1, EPP was used as the independent variable to predict added value of EMR use. Model 2 is a mediation model, in which paths are added that run via the second-order latent construct formal governance, and two measures of culture.

### Analyses

The data are analysed using SEM. SEM, also known as latent variable analysis, combines path-analysis, simultaneous equation models and factor analysis [44, 45]. It enables to estimate associations of latent variables (unobserved variables, measured with multiple items), to estimate multiple regression equations, including more than one dependent variable in one model, and to study whether hypothesized paths are corroborated by the underlying data. The results show how well the items load onto the factors or how well the observed items fit to the latent variables. Next, regression parameters express the strength of the association between the latent variables. The covariances show whether the dependent variables are correlated amongst each other. We used the package ‘lavaan’ in R to conduct the analyses [44].

The evaluation of the fit of the model is presented using three fit measures [46]: the root mean square error of approximation (RMSEA), the comparative fit index (CFI) and the Tucker-Lewis index (TLI). All three have to meet their particular threshold to indicate a good fit. RMSEA values smaller than or equal to 0.05 are considered to be a good fit value [47, 48]; the CFI and TLI need to be 0.90 or higher to indicate a good fit [cf. 48, 49]. Finally, a chi-square/*df* ratio between 2.0 and 5.0 indicates a good model fit [50].

### Sample and data collection

Data were gathered through an online questionnaire that was sent to 2000 doctors and 3623 nurses. The doctors were approached via a panel that specializes in the recruitment of doctors to participate in research. The nurses were approached in a general panel (including a random sample of the population) when they had stated that they worked in a hospital as a nurse. The potential respondents were invited via e-mail to complete the online questionnaire if they worked in hospitals where an EMR was implemented in the period of the data collection. The questionnaire contained items measuring the constructs of the theoretical model (Additional file 1 for the items and scale parameters), questions on personal information and basics information about the work situation. The questionnaire was made accessible through the Internet by an external research agency. After completing the questionnaire, the respondents received a reward. Doctors received a monetary reward, and the nurses were given ‘points’ that enabled them to buy products. According to The Dutch National Ethics Board (Central Committee on Research involving Human Subjects), formal testing by a medical ethical committee was not necessary because (1) the research did not involve medical testing (because it was on healthcare services) and (2) the persons involved were not subjected to actions or behavioural rules.

Four hundred twenty-seven doctors (24 %) and 631 (19 %) nurses were included in the analysis (see Table 1 for sample characteristics). The sample characteristics, age and gender distribution correspond with the national figures on age distribution [51]. However, in our sample, 17 % of the nurses and 24 % of the doctors reported to work in an academic hospital. In the Netherlands, 8 of 85 hospitals are academic (9.4 %) and 90.6 % are general or specialized [51]. This means that in our respondent group, there is an overrepresentation of nurses and doctors from academic hospitals. This can be understood from the fact that we intentionally only included nurses and doctors who had experience with working with EMRs and that academic hospitals in the Netherlands precede the other hospitals in the implementation of EMRs.

**Table 1:** Sample characteristics

	Nurses (631)	Doctors (427)
Mean age (SD)	43.7 (SD = 11.6)	47.6 (SD = 9.6)
Female (%)	79 %	27 %
Position		
Specialist		94 %
Resident		4.5 %
Specialist-assistant not in training		1.5 %
Reported type of hospital		
Academic	17 %	24 %
General	73 %	72 %
Specialized or private	9 %	3 %

### Main latent concepts

In this section, all constructs are presented. The precise items and factor loadings of all items are presented in Additional file 1. We constructed the models based on the theoretical consideration presented in the ‘Background’ section. First, we constructed the scales based on factor analyses. We confirmed the results with reliability analyses, by estimating the Cronbach’s alpha of the scales. Subsequently, we built model 1, to test hypothesis 1. Within the scales, we allowed observed items to covary if this would result in a significant improvement of the model fit (all thetas are reported in Additional file 1). We reasoned that if the model fit would increase when similar items would be allowed to covary, this means that the two items concerned are formulated in such a manner that they measure a similar dimension of the latent construct. To deal with this in these analyses, we allowed a very limited number of items to covary (thetas are reported in Additional file 1), and future studies based on these scales should consider rewording the items to better measure what was intended to be measured. Next, we added the latent variables that we hypothesized to mediate the relation between EPP and added value. Also within these scales, we corrected the model by allowing to estimate error variances of observed variables, based on the same line of reasoning as presented earlier in this paragraph.

#### Success of implementation: added value to the users

There are different means to establish the success of an innovation [e.g. 14, 52, 53]. Here, we focus on the added value of the innovation as perceived by the users. Hence, we apply an individual provider level measure [28] to the introduction of new technologies. As such, it serves as an indicator of organizational success, and we assume it is interrelated with the other measures of successful implementation [52].

Added value as perceived by the users (doctors and nurses) is measured in five items (Additional file 1). The score of this latent variable is higher when users rated to find their EMR to enable them to realize their tasks quicker, improved the quality of their work, made their work easier to do and increased their efficacy at work and their control over work. The factor loadings are presented in Additional file 1. The Cronbach’s alpha is 0.93.

#### Escalation prevention potential

Escalation prevention potential is measured with seven items, which are drawn from previous studies’ organizational mechanisms to limit chances of escalation behaviour [2, 3]. These seven items contain questions about three aspects of innovation projects, namely: (1) the goals of the project, (2) the way in which the process is structured and (3) how the users are supported to be able to use the new technology (see Additional file 1 for exact wording of items). *Goals* are measured with the items asking whether new information technology projects in the organization have a clearly defined aim, whether success factors are formulated and if there are measurable goals. Items about the *process* of the IT projects measure whether such projects consist of predefined stages and whether the project is regularly evaluated. *Ability* is measured by asking whether responsibilities are clearly defined and whether the project participants have sufficient knowledge. The scale represents the respondent’s estimation of the various aspects of EPP. Cronbach’s alpha of this scale is 0.88.

#### Formal governance

Formal governance is a second-order latent variable and is constructed of five other latent variables: support of the administrative department, support of the IT department, support of the HR department, reflexive leadership and bottom-up communication. Each first-order latent variable is presented in the following section. The Cronbach’s alpha of the scale measuring formal governance is 0.77.

##### Support of administrative department

The first dimension of formal governance is the support of the administrative staff regarding entering patient data into the system. If EPP is present in the organization, people at the supportive staff departments can be expected to have sufficient skills and resources to support the staff with the administrative aspects of the EMR and hence to improve the perceived added value of an EMR by its users [34]. The item is measured by three items containing statements on available skills and resources of the administrative department in helping out with problems related to the EMR. Cronbach’s alpha is 0.93.

##### Support of IT department

Following the same line of reasoning as the previous paragraph, EPP is also expected to provide the conditions for an IT department that is well equipped to help the users. Subsequently, the problems that the users face are dealt with swiftly and they will perceive the EMR to have more added value [54]. This latent variable is measured with three items (Additional file 1) indicating available skills and means to support the users of EMR’s. Cronbach’s alpha is 0.91.

##### Support of HR department

Starting to work with an EMR requires new skills and implying the need for education and training during EMR implementation [32]. The HR department may play an important role in evaluating new needs and offering courses and training to facilitate those needs [29] and improve the performance of the clinicians working with the EMRs [33]. Having this in mind, EPP may be expected to lead to a well-thought support of the HR department. This construct is measured with three items (Additional file 1), and the Cronbach’s alpha is 0.90.

#### Reflexive leadership

Besides making sure that the support of other departments is in place, the management of hospitals can differ with regard to the way they communicate. If the managers of an organization engage in reflexive leadership, other organization members legitimize the managers’ role to guide the organization [35–37, 55]. Reflexive leadership is measured with eight items (Additional file 1) and has an Cronbach’s alpha of 0.92.

##### Bottom-up influence

Related to leadership is the extent to which members of the organization can influence decisions of the organization. The level of influence is identified to affect the support of EMR systems [23, 37, 38, 41, 56]. Bottom-up influence is seen here as a part of formal governance, since management can allow and foster this kind of communication. EPP consequently works through this mechanism as a means to have employees voice disagreement, allow for suggested improvements and receive signals of the implementation going wrong, which allow them to intervene. Bottom-up communication is measured in four items, and its Cronbach’s alpha is 0.90.

### Culture

#### Open and innovative culture

Apart from formal governance, culture [38, 39] may explain part of the functioning of EPP. Openness of a culture [29, 41] and an innovative culture [40] may explain part of the relationship between EPP and the success of the implementation. We therefore included two measures of organizational culture in the mediation model, namely: openness of the culture and innovativeness of the culture. Open culture was measured with three items, and its Cronbach’s alpha is 0.73. Innovative culture is measured with three items, and its Cronbach’s alpha is 0.70.

### Control variables

To take into account that responses are affected by background variables of the respondents, a number of control variables are added to the analysis. The control variables are gender (0 = male and 1 = female), age and level of implementation of the EMR. Prior research shows that the implementation stage of the EMRs [22, 57] affects the perception and support of users of EMRs. The measurement of the level of implementation is based on the answers of respondents about how computerized the EMR in their hospital is (or whether it is partly administrated on paper). The score is higher for respondents working with a completely computerized EMR. If the complete EMR was reported to be in one system, the score on this variable is higher (+1), and if the EMR consisted of multiple systems (and not one integrated system), the score is lower (+0.5). And when the data of the nurses were visible for physicians and vice versa, the score of implementation level is higher (+1).

## Results

**Fig. 1:**
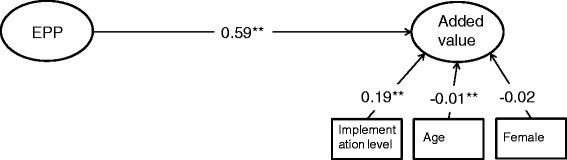
SEM model of hypothesis 1

**Fig. 2:**
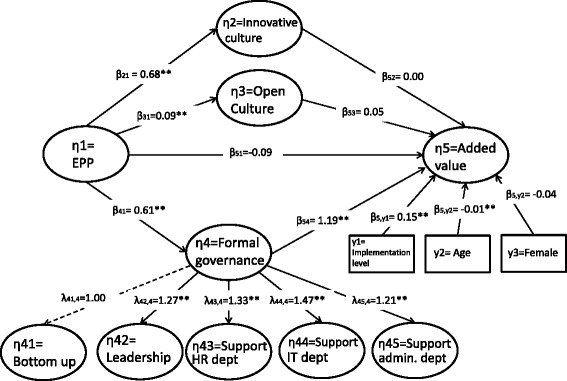
Mediation model with control variables (All-Y notation)

The main results are presented in Table 2 and Figs. 1 and 2. Figure 1 presents the model with EPP as the independent variable and added value as the dependent variable, along with the control variables.

**Table 2:** Regression parameters of main model (model 1) and mediation model (model 2), including control variables, with LISREL, ‘All-Y’ notation

Dependent variable	Independent variables	Model 1		Model 2	
		*β*	SE	*β*	SE
Added value (*η*5)					
	EPP (*β*_51_)	0.59**	0.05	−0.09	0.18
	Formal governance (*β*_54_)			1.19**	0.27
	Open culture (*β*_53_)			0.05	0.08
	Innovative culture (*β*_52_)			0.00	0.07
	Level of implementation (*β*_5,y1_)	0.19**	0.04	0.15**	0.04
	Age (*β*_5,y2_)	−0.01**	0.000	−0.01	0.00
	Female (*β*_5,y3_)	−0.02	0.06	−0.04	0.05
Formal governance (*η*4)					
	EPP (*β*_41_)			0.61**	0.05
Innovative culture (*η*2)					
	EPP (*β*_21_)			0.68**	0.04
Open culture (*η*3)					
	EPP (*β*3_1_)			0.09**	0.02
RMSEA		0.050	0.046		
95 % CI		(0.044–0.056)	(0.043–0.048)		
CFI		0.97	0.95		
TLI		0.97	0.94		
Chi-square (*df*)		299.8 (83)	1637.9 (508)		

** *p* < 0.01

Figure 1 presents the base model. The RMSEA is 0.05 (95 % CI = 0.044–0.056), the CFI and TLI are both 0.97 and the chi-square is 299.8 (*df* = 83) (*p* < 0.01). EPP and added value are positively related: the regression coefficient is 0.59 (*p* < 0.01). This finding is in line with hypothesis 1. The control variables show that older doctors and nurses see more added value in the EMR (*b* = −0.01; *SE* = 0.00) and that the perceived added value is higher, the higher the level of implementation (*B* = 0.19; *SE* = 0.04). In the next steps of the analysis, measures of formal governance and culture are added to investigate whether this relationship changes.

Figure 2 presents the mediation model to investigate whether formal governance and organization culture explain (part of) the association between EPP and added value (model 2 in Fig. 2). The RMSEA of this model is 0.046 (95 % CI = 0.043–0.048), the CFI is 0.95 and the TLI is 0.94. The chi-square is 1637.9 (*df* = 508) (*p* < 0.01). After adding these variables, the regression coefficient of EPP and added value in Fig. 2 is no longer significant (*b* = 0.09; *SE* = 0.18). There are positive and significant relationships between EPP and formal governance (*b* = 0.61; *SE* = 0.05) and between formal governance and added value (1.19, *SE* = 0.27). This outcome corroborates hypothesis 2: formal governance mediates the relationship between EPP and added value.

The coefficients between EPP and innovative culture (*b* = 0.68, *SE* = 0.04) and open culture (*b* = 0.09; *SE* = 0.02) are significant, but the coefficients between added value and innovative culture (*b* = 0.00; *SE* = 0.07) and open culture (*b* = 0.05; *SE* = 0.08) are not. This means that hypothesis 3 is rejected: organizational culture does not explain the relationship between EPP and added value.

## Discussion

In this paper, we studied which organizational factors can explain the relationship between organizational EPP and the successful implementation of an innovation by means of a mediation model. Organizations with more EPP are able to prevent dysfunctional implementation projects and guide the projects towards success. EPP entails a number of organizational checks and balances that have the potential to correct decision-making errors that may occur if innovations are implemented. We found that doctors and nurses who report more EPP in their hospitals were more positive about the added value of their EMR and that this relationship is explained by the formal governance structures supporting the implementation processes. Besides that, we found no evidence that an open or innovative organization culture mediates the relationship between EPP and added value of EMRs for its users.

In assessing these outcomes, we should acknowledge that this study has the following limitations. First, an important question that cannot be answered with this study is whether escalation prevention potential really decreases the likelihood of escalation of commitment. For this study, we focus on the perceived added value by its users, which we used as a proxy of implementation success. This means that the main source of information is one group of stakeholders. If the users find the innovation successful, it can be assumed that they experience benefits from using it. Nevertheless, additional benefits or benefits as perceived by other stakeholders, such as increased organizational efficiency, were not included in the analyses. However, the user’s perception of the added value of the innovation is essential for the other stakeholders to benefit from its potential. The subsequent billing and other administrative and organizational processes rely on the information in the EMR. We therefore assume that perceived added value by its key users is a crucial aspect in measuring the success of an innovation process. However, future research may focus on the multi-dimensionality of success of the implementation processes and aim at answering questions such as: Which dimensions need to be incorporated to get a complete picture? Do stakeholders perceive different dimensions of success? And (how) are the various dimensions related?

Secondly, it should also be noted that the study relies on the perceptions of members of the organization. Although such information is insightful, for example, because organizational members have more confidence in projects if they believe that the managers of their organization can lead them appropriately, it should be acknowledged that these perceptions may not fully capture the escalation prevention potential of an organization.

Thirdly, even though the theoretical model represents explanatory variables, the analyses are based on cross-sectional data. Therefore, causal statements are not allowed and it cannot be excluded that some of the relationships (also) work the other way around. Since project management is a dynamic process, it makes sense to regard the variables in the model as a cycle in which the capacity to prevent escalation also has consequences for routines, leadership reflexivity, employee involvement and support staff quality. This also means that the outcomes should be strictly interpreted in terms of associations and not in terms of cause and effect.

Taking account of these limitations, the results yield some practical implications. We argued that that EPP consists of a number of checks and processes that regulate and correct potential negative spirals. These can be captured in processes and procedures to help managers to critically reflect on their own decisions. We found that hospitals with more EPP were also better equipped in their formal governance: the supporting departments were better equipped to support the employees to work with the EMRs and the employees experienced that their communication was heard and acted upon. Considering the dimensions of formal governance, broadly two types of elements could be distinguished: Bottom-up communication and reflexive leadership measure communicational aspects, emphasizing the relationship between the organization and key users (cf. [58]). The other three aspects focus on the practical support of other organizational elements (the IT, HR and administrative department, respectively). All elements appear to be equally important, suggesting that management should ensure that both the practical support and the more relational aspects of the formal governance in the organization contribute to the organizational EPP. This implies that managers can influence the success of the implementation by tangible managerial improvements, for example, by ensuring constructive two-way communication between the employees and management and by providing sufficient means for departments that support the key users of EMRs.

Contrary to theoretical consideration about organizational culture, the findings concerning the lack of influence of cultural characteristics suggest that changing the organizational culture may not improve the outcome of an innovation, in terms of its added value of the EMR for its users. Two possible reasons may explain why no effect was found. First, organizational culture is a phenomenon that is hard to measure and maybe this study included the wrong empirical measurement to find the relevant cultural aspects in these organizations for these respondents. The measurement of culture in this study measures the personal behaviours concerning trying new work methods and discussing mistakes that are made. These are two person-related aspects, whereas the added value of the EMR is a trade of the innovation. Additionally, the measurements of culture are relation oriented, while the measure of success is more task oriented (similar to the measures of formal governance). A second option may be that the mechanisms that explain why EPP leads to more success in innovation processes, mainly works via formal governance in organizations and open and innovative organizational cultures, contribute little or nothing to the added value for the users. This may imply that other, more task-oriented, cultural dimensions may explain part of the association between EPP and organizational success. Future research may focus on this suggested mechanism.

## Conclusions

Managers implementing measures that increase the EPP of their organizations have made themselves vulnerable to a certain extent: the goals of their project are clearly defined and monitored. The existence of these goals and evaluations of processes shows how well management is doing its job to the other employees in the organization. However, it may be the best way to introduce transparency in the organization and to activate and involve all employees in an organization in realizing its organizational goals. By working according to the principles of EPP, management moves towards a model of rational management, because it acknowledges the dangers related to escalation of commitment: for instance, group think, readjusting goals during the project or denying responsibilities when the project has failed. Management that has equipped its organization with more EPP acknowledges that they are part of the social system in their organization. They are likely to be better connected to their organization and are therefore better able to successfully manage their organization. By acknowledging that social dynamics also exist in management, they are able to enforce the rationality in their decisions during (implementation) projects. By this, they potentially de-escalate commitment and prevent excessive failure of implementation processes in their organizations.

## Additional file

Additional file 1: Latent variables, factor loadings, and wording. (DOCX 235 kb)
