# Supplementary material for: How organizational escalation prevention potential affects success of implementation of innovations: electronic medical records in hospitals
Source: Implement Sci. 2016 May 20;11:75. doi: 10.1186/s13012-016-0435-1 (PMC4875635; doi:10.1186/s13012-016-0435-1)
Supplement: Additional file 1: — Latent variables, factor loadings, and wording. (DOCX 235 kb) [file 13012_2016_435_MOESM1_ESM.docx]

Appendix A Latent variables, factor loadings and wording

| Latent construct | value | s.e. | | | item in questionnaire | | | | | |  |  |  |
| --- | --- | --- | --- | --- | --- | --- | --- | --- | --- | --- | --- | --- | --- |
| **Added value(η5)**  (α=0.94) |  |  | | (1=completely disagree, 5=completely agree) | | | | | | |  |  |  |
| (λ51) covaries with λ55, ψ=0.07** | 1.00 |  | | Enables quicker realization of my tasks | | | | | | |  |  |  |
| (λ52) | 0.87 | 0.03 | | improves the quality of my work | | | | | | |  |  |  |
| (λ53) | 1.00 | 0.03 | | makes working easier | | | | | | |  |  |  |
| (λ54) | 1.06 | 0.03 | | increases my efficacy at work | | | | | | |  |  |  |
| (λ55) covaries with λ51, ψ=0.07 | 0.89 | 0.03 | | increases control over my work | | | | | | |  |  |  |
| **Escalation prevention potential**  **(η1)** (α=0.88) |  | | | When a technological renewal is made…. (1=completely disagree, 5=completely agree) | |  |  |  |  |  |  |  |  |
| (λ11) covaries with λ 12, ψ=0.14** | 1.00 |  | | is the reason of the renewal clear prior to the start | | | | | | | |  |  |
| (λ12) covaries with λ 11, ψ=0.14** | 0.86 | 0.04 | | are success factors appointed prior to the start | | | | | | | |  |  |
| (λ13) | 0.72 | 0.05 | | is the process implemented in phases | | | | | | | |  |  |
| (λ14) | 1.17 | 0.06 | | it is clear who is responsible for the success | | | | | | | |  |  |
| (λ15) | 1.16 | 0.06 | | it is ensured that the staff possesses the necessary skills | | | | | | | |  |  |
| (λ16) | 1.19 | 0.05 | | measurable goals are established prior to the start | | | | | | | |  |  |
| (λ17) | 1.17 | 0.05 | | interim evaluations take place | | | | | | | |  |  |
| **Innovative culture(η2)**  (α=0.64) |  | | | (1=completely disagree, 5=completely agree) | | |  |  |  |  |  |  |  |
| (λ21) | 1.00 | |  | My hospital encourages me to try new ideas that may lead to improved work processes | | | | | |  |  |  |  |
| (λ22) | 0.59 | | 0.04 | If I try to improve a work process, my colleagues are open to it | | | | | |  |  |  |  |
| (λ23) | 0.57 | | 0.04 | My manager will listen if I suggest an idea to improve the work process | | | | | |  |  |  |  |
| **Open culture(η3)**  (α=0.71) |  | |  | (1=completely disagree, 5=completely agree) | | | | | |  |  |  |  |
| (λ31) | 1.00 | |  | If I feel that I did not do my job as I should, I will discuss this with my team members | | | | | |  |  |  |  |
| (λ32) | 2.21 | | 0.21 | If I feel that I did not do my job as I should, I will discuss this with my manager | | | | | |  |  |  |  |
| (λ33) | 1.46 | | 0.11 | Regularly I ask my manager for advice | | | | | |  |  |  |  |
| **Support of administrative department(η45)**  (α=0.93) | The administrative department….. (1=completely disagree, 5=completely agree) | | | | |  |  |  |  |  |  |  |  |
| (λ451) covaries with λ441 of support ITdept, ψ=0.13** | 1.00 | |  | reacts swiftly an adequately when there are problems in registration in the EMR | | | | |  |  |  |  |  |
| (λ452) | 1.12 | | 0.03 | possess the necessary skills and expertise to support proper functioning of the EMR | | | | |  |  |  |  |  |
| (λ453) | 1.13 | | 0.03 | possess the necessary skills and expertise to align the EMR to my work | | | | |  |  |  |  |  |
| **Support of IT department (η44)**  (α=0.91) | The IT department….. (1=completely disagree, 5=completely agree) | | | | |  |  |  |  |  |  |  |  |
| (λ441) covaries with (λ451) of support admin. dept, ψ=0.13** | 1.00 | |  | reacts swiftly an adequately when there are problems in registration in the EMR | | | |  |  |  |  |  |  |
| (λ442) | 1.11 | | 0.03 | possess the necessary skills and expertise to support proper functioning of the EMR | | | |  |  |  |  |  |  |
| (λ443) | 1.11 | | 0.03 | possess the necessary skills and expertise to align the EMR to my work | | | |  |  |  |  |  |  |
| **Support of HR department(η43)**  (α=0.90) | The HR department….. (1=completely disagree, 5=completely agree) | | | | |  |  |  |  |  |  |  |  |
| (λ431) | 1.00 | |  | reacts swiftly an adequately when there are problems in registration in the EMR | | | | | | | | | |
| (λ432) | 1.08 | | 0.03 | possess the necessary skills and expertise to support proper functioning of the EMR | | | | | | | | | |
| (λ433) | 0.99 | | 0.03 | possess the necessary skills and expertise to align the EMR to my work | | | | | | | | | |
| **Reflexive leadership(η42)**  (α=0.92) |  | |  | Management of this hospital….(1=completely disagree, 5=completely agree) | | | | | | | | | |
| (λ421) | 1.00 | |  | engages employees to improve the interaction with others within the hospital | | | | | | | | | |
| (λ422) | 1.04 | | 0.04 | analyses relevant information before making a decision | | | | | | | | | |
| (λ423) | 0.91 | | 0.04 | lets me know how they judge my skills | | | | | | | | | |
| (λ424) | 1.01 | | 0.04 | admits mistakes | | | | | | | | | |
| (λ425) | 1.16 | | 0.04 | listens carefully to various points of view before drawing conclusions | | | | | | | | | |
| (λ426) | 1.07 | | 0.04 | knows when to reconsider her points of view | | | | | | | | | |
| (λ427) covaries with λ428 ψ=0.13** | 0.99 | | 0.04 | encourages everybody to express his or her opinion | | | | | | | | | |
| (λ428) covaries with λ427 ψ=0.13** | 0.99 | | 0.04 | supports the employees | | | | | | | | | |
| **Bottom up influence(η41)**  (α=0.90) | (1=completely disagree, 5=completely agree) | | | | |  |  |  |  |  |  |  |  |
| (λ411) covaries with λ412 ψ=0.41** | 1.00 | |  | I was asked on how the EMR should be implemented beforehand | | | | | | | | | |
| (λ412) covaries with λ411 ψ=0.41** | 1.03 | | 0.03 | Prior to the implementation, I was asked what I needed to do my work better | | | | | | | | | |
| (λ413) | 1.28 | | 0.05 | During implementation I was able to tell what I thought of the implementation | | | | | | | | | |
| (λ414) | 1.24 | | 0.05 | During implementation I was able to tell what I wanted different in the EMR | | | | | | | | | |
| **Formal governance(η4)**  (α=0.77) |  | |  | | | | | | | | | |  |
| (λ41) | 1.00 | |  | Bottom up influence | | | | | | | | | |
| (λ42) | 1.47 | | 0.12 | Support IT department | | | | | | | | | |
| (λ43) | 1.21 | | 0.10 | Support HR department | | | | | | | | | |
| (λ44) | 1.27 | | 0.10 | Reflexive leadership | | | | | | | | | |
| (λ45) | 1.33 | | 0.11 | Support Administrative department | | | | | | | | | |
